# Rapid Systemic Progression After Ureteroscopic Holmium:YAG Laser Ablation for High‐Grade Upper Tract Urothelial Carcinoma in a Solitary Kidney: A Case Report

**DOI:** 10.1002/iju5.70193

**Published:** 2026-05-14

**Authors:** Shinichi Takebe, Nanaho Demizu, Mei Tokumoto, Ryota Morinaga, Taku Mitome, Akitoshi Takizawa

**Affiliations:** ^1^ Department of Urology International Goodwill Hospital Yokohama Kanagawa Japan; ^2^ Department of Urology Yokohama City University Hospital Yokohama Kanagawa Japan

**Keywords:** kidney pelvis, laser therapy, solitary kidney, transitional cell carcinoma, ureteroscopy

## Abstract

**Introduction:**

Endoscopic kidney‐sparing management is increasingly being used for upper tract urothelial carcinoma; however, its use in high‐grade diseases remains challenging.

**Case Presentation:**

An 82‐year‐old man with a solitary left kidney underwent transurethral lithotripsy for left renal and ureteral stones 25 years after right nephroureterectomy for ureteral carcinoma. A 15‐mm papillary tumor was incidentally detected in the upper calyx and partially ablated using a holmium:YAG laser after biopsy. Biopsy revealed a high‐grade urothelial carcinoma. Because the patient had a solitary kidney, radical nephroureterectomy would have necessitated dialysis. Therefore, ureteroscopic laser ablation was performed after counseling. A second laser ablation was performed 2 months later. Twelve days later, imaging revealed an enlarged renal pelvic tumor with multiple metastases. Despite receiving gemcitabine plus carboplatin chemotherapy, the patient died.

**Conclusion:**

Ureteroscopic ablation for high‐grade upper tract urothelial carcinoma may offer limited cancer control and can be followed by extremely rapid systemic progression.

## Keynote Message

Endoscopic kidney‐sparing management may be considered in imperative situations such as a solitary kidney; however, its use in high‐grade upper tract urothelial carcinoma remains challenging. The patient showed extremely rapid systemic progression after ureteroscopic holmium:YAG laser ablation for high‐grade renal pelvic urothelial carcinoma. Clinicians should recognize the limited cancer control with endoscopic ablation in high‐grade diseases and the need for careful patient selection, counseling, and surveillance.

**Abbreviations**
CT: computed tomography
KSS: kidney‐sparing surgery
TUL: transurethral lithotripsy
UTUC: upper tract urothelial carcinoma

## 1 Introduction

Endoscopic kidney‐sparing surgery (KSS) is primarily recommended for low‐risk upper tract urothelial carcinoma (UTUC), while radical nephroureterectomy remains the standard approach for high‐risk diseases [1, 2, 3]. Nevertheless, KSS may be considered in imperative situations to preserve renal function, such as in solitary kidney or advanced chronic kidney disease [4, 5, 6]. However, kidney‐sparing therapy for high‐grade or high‐risk UTUC remains challenging because of the substantial risk of recurrence and progression [7, 8, 9].

## 2 Case Presentation

An 82‐year‐old man was referred to our hospital because of left renal and ureteral stones detected on computed tomography (CT) (Figure 1). He had undergone right nephroureterectomy for ureteral carcinoma 25 years prior, leaving a solitary left kidney. Three years prior, urinary cytology had been class III; however, CT at that time showed no upper urinary tract or bladder lesions and he was observed. At presentation, serum creatinine was 1.37 mg/dL and estimated glomerular filtration rate was 38.8 mL/min/1.73 m^2^.

**FIGURE 1:**
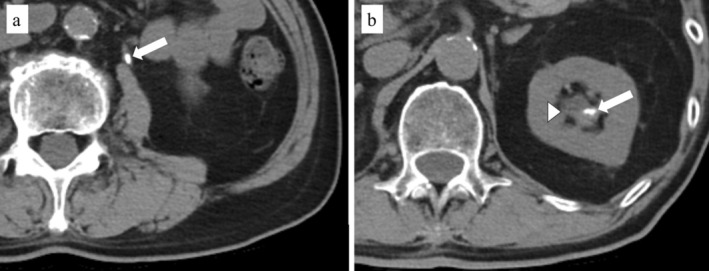
Non‐contrast CT before ureteroscopic surgery. (a) Left ureteral calculus (arrow). (b) Left renal calculus (arrow). A subtle high‐density lesion with a fluid level is visible in the upper calyx (arrowhead), which was retrospectively considered to correspond to a papillary tumor that was later identified during ureteroscopy.

Extracorporeal shock wave lithotripsy for the left ureteral stone failed and was complicated by post‐renal acute kidney injury (serum creatinine, 3.60 mg/dL). A double‐J ureteral stent was inserted and renal function improved.

After recovery, the patient underwent left transurethral lithotripsy (TUL) using a flexible ureteroscope, 10/12‐Fr ureteral access sheath, and holmium:YAG laser (VersaPulse Select 80; Boston Scientific, USA). Irrigation was performed using a pressurized foot‐pump system (Peditrol Unit; Takai, Tokyo, Japan), although the pressure was not recorded. Intraoperatively, a 15‐mm papillary tumor was incidentally detected in the upper calyx. A retrospective review of the preoperative CT showed a high‐density lesion with a fluid level in the same calyx, initially interpreted as blood but later considered to correspond to the tumor (Figure 1). Stone fragmentation was completed, followed by tumor biopsy using partial laser ablation during the same session, as immediate tumor reduction was considered beneficial for this patient. A double‐J ureteral stent was inserted after the first procedure. The operative time was 68 min. The patient developed acute pyelonephritis postoperatively, was treated with antibiotics, and was discharged on postoperative Day 7.

Biopsy revealed a high‐grade urothelial carcinoma, and the depth of invasion was indeterminate. Radical nephroureterectomy was recommended; however, dialysis was inevitably required because the patient had a solitary kidney. After counseling regarding the limited likelihood of durable cancer control and risk of disease progression, kidney‐sparing endoscopic management was pursued.

After recovery from postoperative pyelonephritis and pathological confirmation of high‐grade urothelial carcinoma, a second ureteroscopic laser ablation was performed 2 months later using the same access sheath and holmium:YAG laser. In addition to the upper calyceal lesion, two small papillary lesions were observed in the renal pelvis. All visible tumors were ablated using laser settings of 0.5 J and 10 Hz. A double‐J ureteral stent was placed after the second laser ablation procedure. The operative time was 37 min. No immediate peri‐operative complications were observed.

Twelve days after the second laser ablation, CT performed at another hospital incidentally revealed multiple pulmonary nodules. Positron emission tomography‐CT was performed immediately. Examination revealed enlargement of the left renal pelvic tumor and multiple metastases involving the lymph nodes, both lungs, the liver, and bones (Figure 2).

**FIGURE 2:**
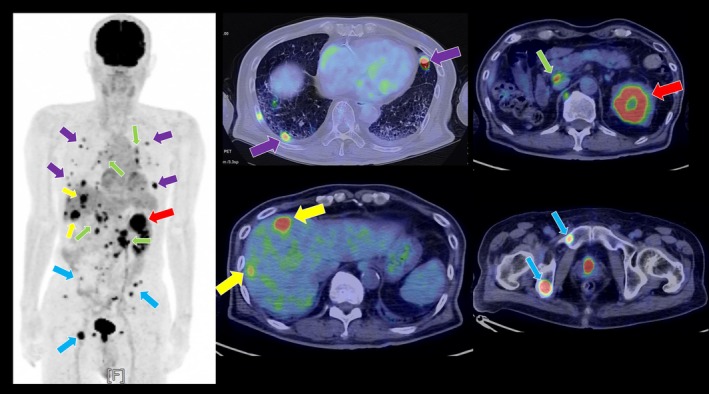
Positron emission tomography‐CT at the time of metastatic diagnosis. The primary left renal pelvic tumor is indicated by a red arrow. Metastatic lesions are indicated by green (multiple lymph node metastases), purple (multiple pulmonary metastases), yellow (multiple liver metastases), and blue (multiple bone metastases) arrows.

Gemcitabine plus carboplatin therapy was initiated soon after the diagnosis of metastatic disease. His general condition gradually deteriorated, and contrast‐enhanced CT after the first cycle showed progression of both the primary tumor and metastatic lesions. Because of the marked decline in performance status and overall condition, second‐line systemic therapy was not initiated, and the patient died approximately 3 months after the metastatic disease was detected. The patient's clinical course is summarized in Figure 3.

**FIGURE 3:**
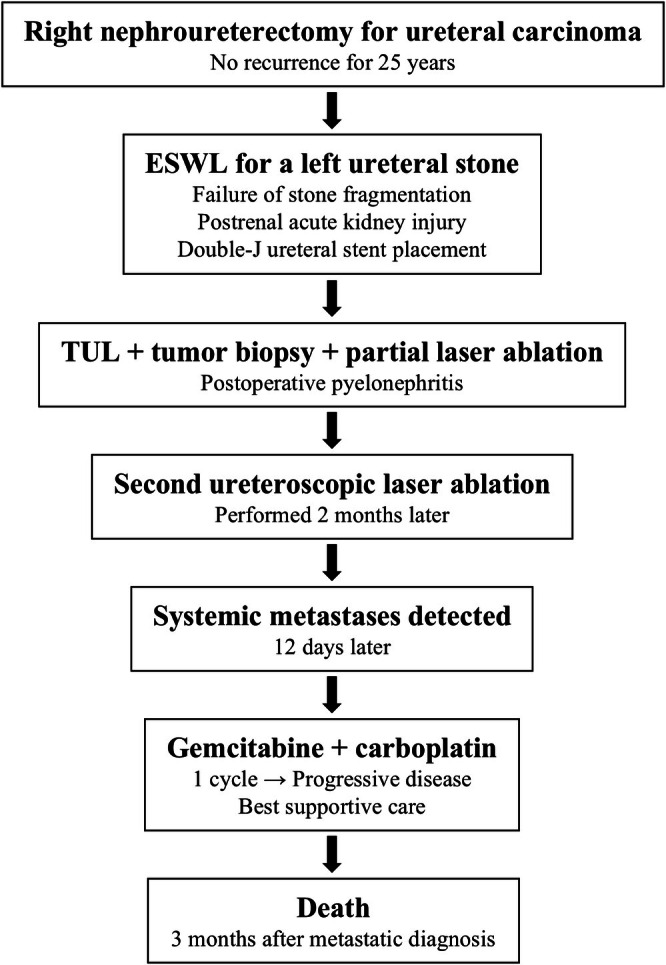
Clinical timeline of the present case. The patient's clinical course included prior right nephroureterectomy, extracorporeal shock wave lithotripsy for a left ureteral stone, initial transurethral lithotripsy with biopsy and partial laser ablation, postoperative pyelonephritis, second laser ablation, diagnosis of systemic metastases, systemic chemotherapy, and death.

## 3 Discussion

Guidelines consistently recommend KSS primarily for carefully selected low‐risk UTUC, while radical nephroureterectomy remains the standard approach for high‐risk diseases [1, 2, 3]. Although risk stratification specifically for endoscopic laser ablation has not been fully established, guidelines incorporate tumor grade, tumor size, and multifocality. The European Association of Urology guidelines emphasize tumor grade as a strong criterion for defining high‐risk diseases [1]. In a cohort treated with flexible ureteroscopy and holmium:YAG laser ablation, progression‐free survival did not differ according to tumor size or tumor distribution, whereas high‐grade histology significantly worsened progression‐free survival and was the only independent predictor of progression [8]. In imperative situations, such as a solitary kidney or advanced chronic kidney disease, endoscopic management may preserve renal function; however, oncologic outcomes for high‐grade disease are generally inferior to those with radical surgery [4, 5, 6, 7, 8, 9, 10, 11].

This case has two clinically important features. First, it represents metachronous contralateral UTUC diagnosed 25 years after nephroureterectomy for unilateral ureteral carcinoma. In a large population‐based cohort, metachronous contralateral UTUC occurred in 0.6% of patients, and approximately 90% of the contralateral tumors were diagnosed within 10 years of the initial UTUC [12]. Although rare, this case suggests that contralateral UTUC may occur even after a prolonged disease‐free interval and that long‐term vigilance may be warranted.

Second, the systemic course was strikingly aggressive. Disseminated metastases were recognized only 12 days after the second ablation, and death occurred within approximately 3 months. A recent scoping review and our targeted search did not identify comparable reports of explosive systemic progression immediately after ureteroscopic ablation [7].

Although causality cannot be proven, the temporal sequence raises the possibility that the initial ureteroscopic intervention contributed to the hematogenous dissemination. In retrospect, biopsy alone followed by staged treatment may be a more cautious strategy than immediate partial ablation during the initial procedure. One possible mechanism is the increased intrarenal pressure during ureteroscopy. Elevated intrarenal pressure has been associated with infectious complications, including urosepsis [13, 14], and in the present case, the combination of a 10/12‐Fr ureteral access sheath, pressurized foot‐pump irrigation, and postoperative pyelonephritis suggests that a pressure‐related backflow may have occurred. This backflow could theoretically facilitate tumor cell dissemination.

Postoperative pyelonephritis may also promote a proinflammatory microenvironment that favors tumor progression and dissemination [15]. Although this mechanism was not confirmed in the present case, it may represent another possible explanation for the unusually aggressive clinical course of the disease.

Despite the use of a holmium:YAG laser in the present case, the current evidence remains insufficient to support the superiority of any specific laser type for the endoscopic management of UTUC [16, 17]. At our institution, ureteroscopic laser ablation for UTUC is performed in approximately 10 cases per year. Nevertheless, the absence of ureteroscopic still images and intraoperative video documentation is a limitation of this study.

Overall, this case reinforces the fact that high‐grade UTUC has limited curative potential with endoscopic ablation and requires cautious patient selection, counseling, and surveillance.

## 4 Conclusion

Endoscopic holmium:YAG laser ablation for high‐grade UTUC may provide limited durable cancer control and can be followed by extremely rapid systemic progression. When nephroureterectomy is not feasible, patients should be counseled about the oncologic risks and require close surveillance.

## Consent

Informed consent was obtained from the patient and his family for the publication of this case report.

## Conflicts of Interest

The authors declare no conflicts of interest.

## Data Availability

The data that support the findings of this study are available on request from the corresponding author. The data are not publicly available due to privacy or ethical restrictions.
